# Cardiovascular Events throughout the Disease Course in Chronic Myeloid Leukaemia Patients Treated with Tyrosine Kinase Inhibitors—A Single-Centre Retrospective Study

**DOI:** 10.3390/jcm9103269

**Published:** 2020-10-12

**Authors:** Andreea Varga, Ioan Tilea, Dorina Nastasia Petra, Mariana-Cornelia Tilinca, Mirela Liana Gliga, Smaranda Demian

**Affiliations:** 1 Department ME2, Faculty of Medicine in English, “G. E. Palade” University of Medicine, Pharmacy, Science and Technology of Targu Mures, 540142 Targu Mures, Romania; andreea.varga@umfst.ro (A.V.); mariana.tilinca@umfst.ro (M.-C.T.); 2 Department of Cardiology II, Emergency Clinical County Hospital, 540042 Targu Mures, Romania; ioan.tilea@umfst.ro; 3 Department M4, Clinical Sciences, Faculty of Medicine, ”G. E. Palade” University of Medicine, Pharmacy, Science and Technology of Targu Mures, 540142 Targu Mures, Romania; mirela.gliga@umfst.ro; 4 Department of Internal Medicine II, Emergency Clinical County Hospital, 540042 Targu Mures, Romania; 5 Department of Diabetology, Emergency Clinical County Hospital, 540136 Targu Mures, Romania; 6 Department of Nephrology, Clinical County Hospital, 540072 Targu Mures, Romania; 7 Department M3, Clinical Sciences Internal Medicine, Faculty of Medicine ”G. E. Palade” University of Medicine, Pharmacy, Science and Technology of Targu Mures, 540142 Targu Mures, Romania; smaranda_demian@yahoo.com; 8 Department of Hematology, Emergency Clinical County Hospital, 540136 Targu Mures, Romania

**Keywords:** chronic myeloid leukaemia, tyrosine kinase inhibitors, cardiovascular risk, comorbidities

## Abstract

Introduction. Cardiovascular risk factors, pre-existing comorbidities, molecular factors, and the direct effects of second- and third-generation BCR-ABL1 tyrosine kinase inhibitors on the vascular endothelium contribute to the progression of cardiovascular (CV) events, especially atherothrombotic conditions. The study objective was to evaluate comorbidities, the cardiovascular risk profile, and events throughout the chronic myeloid leukaemia disease course. Methods. Retrospective data from adults who experienced haematology treatment at a single centre were continuously updated and followed throughout the disease course. A total of 43 subjects conforming with the inclusion and exclusion criteria of the study protocol were finally recruited. The median disease course was 77.0 ± 17.5 months. Statistical analyses were performed. Results. More than three CV risk factors were identified in 41.9% of cases. Almost half of the cases had relevant comorbidities (Charlson Comorbidity Index (CCI) ≥ 4), and no statistically significant comorbidities were found when comparing the tyrosine kinase inhibitor (TKI) treatment subgroups (*p* = 0.53). The patients at high and very high CV risk, according to Systematic Coronary Risk Evaluation (SCORE) risk classification, had 75.0% CV events (12/22 patients), *p* = 0.45. Throughout the disease course, 19 cardiovascular events were reported in 37.2% patients (13 males/3 females, *p* < 0.03). Conclusion. To the best of our knowledge, this is the first study exploring cardiovascular risk factors in Romanian chronic myeloid leukaemia patients. This study reinforces the need for close long-term follow-up that should be performed by a multidisciplinary team. The target should be not only the disease and specific drug-related toxicities but, also, the identification of cardiovascular and metabolic risk factors before the commencement of and throughout TKI therapy.

## 1. Introduction

Chronic myeloid leukaemia (CML) is a clonal myeloproliferative disease whose incidence increases with age. The current prevalence is estimated to be 1.75–12/100,000 inhabitants, with CML patients having a trend of increasing life expectancy comparable to the general population [1,2]. Currently approved tyrosine kinase inhibitors (TKIs) have improved the CML treatment and outcome, and most patients experience a durable therapeutic response. Each one of the approved drugs (imatinib, dasatinib, nilotinib, bosutinib, ponatinib) used in the CML treatment has known adverse drug-specific effects frequently associated with the second generation of TKIs. About one-tenth of patients do not tolerate TKIs; the onset of potential cardiovascular TKI-related toxicities is variable and particularly mechanism-dependent, appearing early after starting the therapy or during treatment [3,4].

Recent studies suggest that cardiovascular (CV) risk factors, pre-existing comorbidities, and molecular factors, along with the direct effects of the second- and third-generation BCR-ABL1 tyrosine kinase inhibitors on the vascular endothelium, together contribute to the progression of CV events, especially atherothrombotic conditions [5]. Ischaemic heart disease, cerebrovascular events, and lower extremity arterial disease have a reported incidence ranging between 2% and 5%, being more frequent in patients treated with nilotinib and ponatinib compared with imatinib- and dasatinib-treated patients [6]. Hypertension (HTN) is regularly issued in cancer registries; the incidence of TKI-induced HTN varies considerably among different TKI regimens from 7.8% of 818 patients treated with bosutinib [7] to 68% of 449 patients with ponatinib [8]. TKI-induced HTN is also related to dose and tumour type. Previous studies described the association between long-term TKI therapy, mainly dasatinib, and distinctive respiratory side effects such as dyspnoea, development of pulmonary arterial hypertension (PAH), exudative pleural effusion, and pulmonary parenchymal abnormalities [9]. The precise underlying molecular mechanisms of dasatinib-induced PAH are currently unclear; one proposed mechanism is linked to the Src family kinase pathway [10].

The 10-year cardiovascular risk of CML patients can be assessed using the Systematic Coronary Risk Evaluation (SCORE) chart, Framingham score, or QRisk^®^3–2018 algorithm; thus, the precondition of atherosclerotic risk events in patients, before and during treatment with TKIs, should be evaluated [11,12,13,14,15].

The objective of our study was to evaluate the cardiovascular profile, comorbidities, and cardiovascular events throughout the disease course in CML patients treated with tyrosine kinase inhibitors.

## 2. Materials and Methods

In a single centre of adults who experienced haematology treatment, we identified a cohort of 51 CML patients (aged between 22 and 81 years) that followed the European CML diagnostic criteria [16]. A total number of 43 subjects, both male and female, conforming with the inclusion and exclusion criteria for the study protocol, were recruited.

A retrospective analysis of patient characteristics at diagnosis time, continuously updated and followed in the time of disease course, in addition to specific results of patient re-evaluation, completed between January 2019 to June 2019, is presented.

The median disease course was 77.0 ± 17.5 months (IQR 10–228 months).

At the time of retrospective analysis, 32 patients were treated with the first-line TKI treatment. Second-line TKI treatment was initiated in 11 patients at different moments throughout the disease course due to suboptimal response or intolerance to imatinib.

Sociodemographic data, past medical history elements suggestive for concomitant CV diseases, and ongoing treatments were collected and recorded from discharge papers and electronic medical databases. Baseline comorbidities of interest—cigarette smoking, obesity, lipid disorders, diabetes mellitus (DM) status, chronic kidney disease (CKD), and several concomitant diseases—were also registered.

All study subjects were monitored for "de novo” onset of cardiovascular and atherothrombotic (AT) events. Cardiovascular events were defined as hypertension, heart failure, presence of documented arrhythmias, or QT prolongation and abnormal echocardiography. Atherothrombotic events included acute coronary syndromes, and cerebrovascular and lower extremity artery disease (LEAD) events. [17,18]. 

Complete blood count (Sysmex XS-1000i^TM^, Sysmex Europe GmbH, Norderstedt, Germany), plasma glucose, lipid panel, blood urea nitrogen (BUN), and creatinine (Konelab Prime 60i, Thermo Fisher Scientific Inc., Waltham, MA, USA) were determined from fasting venous blood samples at diagnosis and different moments of disease course. For staging chronic kidney disease, values of estimated glomerular filtration rate (eGFR) were used (CKD-EPI formula) [19].

Baseline and, at least annually, 12-lead electrocardiograms were recorded for the entire cohort (BTL-08 Plus machine, BTL Industries, Hertfordshire, UK). At the time of diagnosis, documented history of myocardial ischaemia associated with consistent ECG changes were considered in the diagnosis of the chronic coronary syndrome. Cardiac dysrhythmias and QT interval prolongation were also evaluated (medical history, including certain medications that can prolong QT interval). The Bazett formula was used to assess the QT calculated interval (QTc = QT/√RR).

Hypertension history and heart failure (HF) diagnosis were collected. The study team reassessed all patients at the time of the retrospective analysis moment according to current guideline recommendations [20,21]. During the follow-up period, uncontrolled HTN (≥140/90 mmHg) was confirmed by at least two separate office blood pressure measurements using a validated device (Omron M3 Intellisense, Omron Healthcare Co. Ltd., Japan).

Left ventricular systolic function (left ventricular ejection fraction—LVEF), systolic pulmonary artery pressure, and the potential presence of pericardial effusion were reassessed using a GE Vivid^TM^E9 machine (GE Healthcare, Chicago, IL, USA). A tricuspid regurgitation peak gradient (TRPG) value higher than 31 mmHg was considered as indicating systolic pulmonary hypertension (PH) [22].

All electrocardiograms and echocardiography data were blind-reviewed by expert cardiologists.

Determination of the ankle–brachial index (ABI) was routinely performed in treated high-risk cases. Suspected LEAD was evaluated by duplex ultrasonography; computed tomography angiography was used to assess stenotic/occlusive disease [23].

The 10-year risk of fatal cardiovascular disease (CVD) was calculated using the Systematic Coronary Risk Evaluation (SCORE) [24]. The high-risk chart was applied to data extracted at the time of diagnosis for each patient.

Sokal, Hasford, and ELTS scores were assessed at the diagnosis time [25]. Age-adjusted risk of death estimation related to concomitant diseases was also carried out using the Charlson Comorbidity Index (CCI) [26,27].

In accordance with the rules of the Helsinki Declaration and International Ethical Regulations, all entrants provided written informed consent. This study was conducted under the approval of the Targu Mures University of Medicine and Pharmacy Research Ethics Committee (approval number 170/2018).

## 3. Statistical Analyses

Statistical analyses were accomplished using Microsoft Excel© version 16.36 spreadsheets (Microsoft^®^ Office 2020; Microsoft Corporation, Redmond, WA, USA), GraphPad Prism^TM^ 8.4.3 (471) Software (GraphPad Software Inc, San Diego, CA, USA), and SPSS statistical software (SPSS statistics for Windows, Version 27.0. Armonk, NY: IBM Corp.). In basic statistics, descriptive, qualitative data were presented as counts and percentages. Regarding the relatively small sample of our study, the association between qualitative variables was assessed using the chi-squared and Fisher’s exact test in the analysis of contingency tables. Quantitative data are presented as mean and standard deviation (for normally distributed data). Non-parametric data were obtained using the Mann–Whitney and Kruskal–Wallis tests. In an exploratory analysis, Cox proportion hazards regression models were built to test the interaction of CV/AT events and effusion events, with the SCORE risk stratification, traditional CV risk factors (body mass index, HTN, DM, familial history of CV disease), treatment regimen, and therapeutic response. For all tests, statistical significance was set at *p*-value < 0.05, and the confidence interval was 95%. 

## 4. Results

The gender distribution, male-to-female ratio, was 1.52/1 (26 males) with the cohort mean age of 56.3 ± 15.0 years (range 22–81 years). Using the obesity WHO classification, we identified 34.9% regular-weight patients, 34.9% overweight, and 30.2% obese patients at the time of confirmation of CML diagnosis. Hypertension was the most common comorbidity, affecting 20/43 patients (46.5%, 9 males and 11 females).

The patient characteristics at the time of diagnosis are depicted in Table 1.

Patient profile in terms of mean haematological and biochemistry data before the first treatment line administration are displayed in Table 2.

Before TKI therapy administration, the distribution of CV risk factors in patients ranged between 9.3% (4 patients) with no risk factors, up to 18.6% patients (8 cases) presenting one risk factor; 30.2% patients had a minimum of two risk factors (13 subjects). More than three CV risk factors were identified in 41.9% (18 pts, 11 males, 7 females).

Analysing the comorbidities at the time of CML diagnosis, a CCI score ≥ 4 was reported in almost one half (48.8%) of the cases. The most common CCI-relevant comorbidities were: moderate CKD (6 cases), diabetes mellitus (6 patients), congestive HF (5 cases), myocardial infarction (2 cases), chronic pulmonary obstructive disease (2 patients), peptic ulcer disease (2 subjects), LEAD, dementia, and mild liver disease (1 case each). There was no statistical significance comparing the TKI treatment indication regimen (*p* = 0.53) (see Table 3).

The comparison of patients’ cardiovascular SCORE risk linked to different TKI regimens at the time of diagnosis and throughout disease course is presented in Table 4; at the diagnosis time, 39.5% patients presented a very high CV SCORE risk.

In the descriptive analysis of patient characteristics reassessed following the median disease course of 77.0 ± 17.5 months, high levels of cholesterol and increased systolic and diastolic blood pressure (SBP and DBP) were observed. The corresponding data are displayed in Table 5.

Throughout the disease course, with respect to the study cohort, 19 CV/AT events were reported in 16 patients (13 males/3 females), 37.2% of all cohort, (*p* = 0.03).

The patients in the high and very high CV SCORE risk classification had 75.0% CV events (12/22 patients), *p* = 0.45.

The detailed CV/AT events and appearance of pericardial/pleural effusions, reported during the CML therapy, are as follows: 8 CV/AT events and 4 effusions in 11 patients on imatinib; 6 CV/AT events and 3 effusions (9 patients) during nilotinib treatment; 5 CV and AT events, 5 effusions were experienced by 5 patients during dasatinib treatment (Table 6).

The assessment of cardiovascular events throughout the disease course identified three cases experiencing an acute myocardial infarction (STEMI). Two high-risk CV patients were identified with different TKI regimens, with one case for imatinib and one case for dasatinib treatment.

One of the high-risk CV cases was a 70-year-old man on a daily regimen of 400 mg imatinib for 18 months (started in 2012) who then switched to 100 mg dasatinib o.d. In 2016, under the dasatinib regimen, he presented a STEMI, and primary percutaneous coronary intervention was successfully performed.

The third case developed STEMI six years (2018) after CML diagnosis. A 38-year-old male with low CV risk was diagnosed with CML in January 2012. He underwent uninterrupted daily treatment with 400 mg imatinib until 2014, then switched to 800 mg nilotinib daily. At the time of the acute CV event, the immediate invasive strategy was followed by stent implantation. Secondary to this event, TKI was withdrawn, and treatment with interferon and hydroxyurea was initiated. The patient was referred for medullar transplantation.

A single ischaemic stroke event was recorded in a 76-year-old man with a known 10-year history of HTN. First-line treatment, with a duration of nine years (2008 to 2017), was imatinib 400 mg o.d., which then switched to a second-line treatment of nilotinib 400 mg b.i.d. with a major molecular response (BCR-ABL1 ≤ 0.1%). Following 28 months of continuous nilotinib treatment, the patient was admitted to the neurology department where left hemiparesis, hemihypoesthesia, and central facial palsy were observed. A magnetic resonance imaging scan revealed multiple acute ischaemic subcortical lesions in the right frontal lobe and lacunar infarctions in the right midbrain, thalamus, and caudate. Nilotinib was withdrawn, and antiplatelets associated with lipid-lowering agents were prescribed.

One case of confirmed, complicated LEAD was identified in a 70-year-old patient from the nilotinib regimen, with a long-standing history of CML (11.6 years) and first-line imatinib for seven years. A notable history of concomitant CV personal risk factors (HTN, obesity, dyslipidaemia, ex-smoker), besides a familial history of significant CV disease (myocardial infarction), were previously described. Medical management included limited surgery (single finger amputation) and conservative treatment; nilotinib was discontinued, and bosutinib was prescribed.

Within a mean disease course of 77 months for the entire study cohort, in 2019, echocardiographic studies were performed within a time frame of 6 months (January–June). A mean TRPG value of 19.8 mmHg (range 14–34 mmHg) for the imatinib regimen, 25.9 mmHg (range 16–32 mmHg) for nilotinib, and 26.0 mmHg (IQR 15–42 mmHg) in patients treated with dasatinib was noted. Two patients presented TRPG values higher than 31 mmHg, suggesting the presence of dasatinib-induced PH, but neither underwent invasive measurement of the pulmonary artery mean pressure by right heart catheterisation. Pulmonary embolism was ruled out by computed tomography and laboratory tests for rheumatoid factor, anti-nuclear antibodies, and Scl-70 were negative. A 57-year-old man, diagnosed with CML in 1999 and continuously taking 400 mg imatinib daily for 14 years, then changed to 100 mg dasatinib o.d. (2013–present), was identified with a TRPG of 38 mmHg and no apparent LV systolic dysfunction or any asynergy of LV wall motion; LVEF was 60%. Dasatinib-induced PH was suspected.

One of the STEMI cases in which the patient had a stable cardiovascular status one year after the acute CV event complained of limited respiratory symptoms (dyspnoea). The echocardiography examination revealed a TRPG of 42 mmHg, apparent LV systolic and diastolic dysfunction, an LVEF of 55%, and no LV wall motion asynergy. Dasatinib-related dyspnoea was suspected, and temporary discontinuation of TKI was indicated. After one month free of dasatinib, the CML treatment was restarted. Bosutinib was initiated without short-term adverse effects.

Seven cases were newly diagnosed with hypertension. Treatment intervention for uncontrolled blood pressure was performed in 24 patients: 11 on imatinib, 9 on nilotinib, and 4 on dasatinib at the re-evaluation time, compared to 8 cases at the time of CML diagnosis (*p* = 0.0007). Seventeen new uncontrolled HTN cases, in which HTN was previously under control, were identified (*p* = 0.05).

No QT interval prolongation was noted during the TKI treatment in the study cohort.

The pericardial/pleural effusion appearance reported during the TKI regimen ranged between 18 to 114 months (54.7 ± 32.1 months) and had a total incidence of 27.9%, with the highest incidence (55.5%) in the dasatinib-treated patients; in the Cox multivariate regression model, dasatinib is a positive predictor factor (HR, 1.6; 95% CI, 0.7–3.7) for effusion.

In our study, the multivariate Cox regression model of SCORE risk stratification, CV risk factors, and therapeutical response displayed none of the covariables as significant predicted factors for the incidence of CV/AT events. Assessing the effusion appearance events in the multivariate Cox regression model, there was a negative association with SCORE risk stratification (HR, 0.8; 95% CI, 0.4–1.4), and with the family history of positive CV disease (HR, 0.4; 95% CI, 0.1–1.9). The CV event probability displayed higher association with renal failure, although in our study this was not significant (*p* > 0.05; HR, 0.5; 95% CI, 0.2–1.4).

The therapeutical response as a predictor effect for effusion events, in time-dependent Cox regression exploratory analysis, is almost significant (*p* = 0.059). For patients who failed to respond to TKI therapy, the hazard of effusion appearance increases by 1.4%. 

## 5. Discussion

The natural history of CML dramatically changed secondary to the development of the TKIs. Most TKIs are relatively well tolerated, but each of them has a distinct toxicity profile for which patients should be monitored [28].

Disease-specific survival has improved significantly, so controlling comorbidities and minimising treatment toxicity should be a priority for the clinician. Cardiovascular risk factors need to be recognised and appropriately managed as second-generation TKIs demonstrate higher CV risk compared to imatinib. Cardiovascular comorbidities influence CML patients’ survival. Cardiovascular events mainly develop in elderly patients with CV risk factors [29].

Our study focused on identifying CV events and risk factors during treatment with TKIs in real-life practice.

According to our analysis, the majority of CV events occurred in very high CV-risk patients. Thus, we consider that CV risk should be investigated when initiating TKI treatment. Reversible risk factors should also be considered.

In a study by Breccia et al., the incidence of CV risk evaluated with SCORE was increased for high-risk patients, and there were no cardiovascular events in low-risk patients [27].

Osada et al. suggested a relationship between CV risk assessed with SCORE chart and predicting molecular response to TKIs [30].

Experimental data and clinical trials of imatinib have shown a tolerable safety risk profile with no increase in atherosclerotic events; in fact, it might even prevent them compared to new-generation TKIs [31,32]. Nilotinib has pro-atherogenic and anti-angiogenic potential on the vascular endothelium and an effect on platelet activation and thrombus formation compared to imatinib and dasatinib [33,34].

Higher rates of myocardial infarction were reported by Dahlen et al. in patients treated with nilotinib and dasatinib compared with imatinib, 84% of whom had at least one major cardiac risk factor [35]. Accordingly, in a retrospective study in a real-world setting by Gora-Tybor et al., vascular events were reported in 4% of patients treated with dasatinib and 11% of patients treated with nilotinib. Two of the patients receiving nilotinib died, one from myocardial infarction and one from ischaemic stroke [36]. Quintás-Cardama et al. identified five vascular events, but risk factors for vascular disease were present in only one patient [37].

In a recent paper published by Novo et al., it was suggested that the latest generation of TKIs used in the treatment of CML (nilotinib, dasatinib, and ponatinib) may increase the incidence of CV events; they also observed pleural effusions [38]. In our study, 8/43 patients (18.60%) treated with nilotinib and dasatinib developed pericardial effusion, and two of them presented moderate pleural effusion.

Levato et al. observed a 14.8% incidence of progressive LEAD and other vascular events during nilotinib therapy [39]. The probability of remaining free of progressive LEAD is higher in patients treated with imatinib compared to those with nilotinib regimens [38].

Cardiovascular events are associated with CV risk factors, and prospective studies are needed to establish patient risk profiles [40]. Hyperlipidaemia and hyperglycaemia are major risk factors for CV disease and are associated with nilotinib therapy [41]. Homogenous management is essential to minimise the risk of related CV events. Incorporating cardiac biomarker measurement in current settings should be considered in CV risk assessment, surveillance protocols, and workup. In our study, CV risk factors (SCORE risk stratification, BMI, DM, positive history of CV disease) were not significant predicted factors for the incidence of CV/AT events.

Changes in eGFR, congestive HF, chronic coronary syndromes, and chronic obstructive pulmonary disease are associated with the risk of having CV events [42]. In our study, the renal function alteration was also associated with the CV event probability. 

Our study showed an overall incidence of 37.2% CV/AT events in 12 of 43 cases. The incidence of CV events was 4.29% in patients with CML in a study performed in Brazil on 233 patients, being more frequent in patients with second-generation TKIs. Arterial hypertension, dyslipidaemia, CAD, heart failure, and CKD have been associated with an increased risk of CV events. Patients treated with nilotinib and with high- and very high-risk CV (SCORE chart) were noted to have more frequent arterial events [43].

Treatment of CML is evolving. A new therapeutic option could involve a combination of imatinib and chemical compounds capable of exerting antiproliferative effects against K562-resistant leukaemia cells via PPARγ activation. In this direction, sartans, especially telmisartan, have been extensively studied in recent years [44,45,46]. This is an intriguing hypothesis that can also have a potential benefit on the comorbidity treatment (HTN, HF) of CML patients as it is known that the therapeutic plasmatic concentration of telmisartan can be assessed during administration [47].

Recommended therapeutic options for patients with very high CV risk are imatinib and dasatinib [48].

Given the average life expectancy of patients with optimal response to therapy, we believe that the individual risk of the patient should be reduced by optimal selection of TKIs. The primary care physicians who routinely monitor patient comorbidities can detect CV events, possible treatment failure, or disease progression, and must ensure that the patient is seen by the haematologist for evaluation [49].

The most common parameters of the CCI in our research were moderate CKD and diabetes mellitus. Other underlying diseases (congestive heart failure, myocardial infarction, chronic pulmonary obstructive disease, peptic ulcer disease) were key elements of the CCI. Appropriate management of concomitant diseases in cancer patients is required in order to improve health status and quality of life. In CML patients, irrespective of the presence of comorbidities, CCI is a practical tool to assess long-term prognosis, especially in older patients [2]. In conclusion, having concomitant diseases at the moment of diagnosis is associated with poor outcome in CML patients treated with TKIs, and outcome is better predicted by the CCI than Sokal and Hasford scoring.

The study has inherent limitations related to a relatively small number of patients from a single healthcare centre, it being known that CML has a low incidence, and analysis of a larger cohort can possibly offer more robust data. Another shortcoming is that precapillary pulmonary hypertension cannot be confirmed by right heart catheterisation. Underestimation of some comorbidities due to the variations in codification practice can be avoided by using dedicated, specific questionnaires. Monitoring medical education should be a goal in our country.

## 6. Conclusions

To the best of our knowledge, this is the first study exploring CV risk factors in Romanian chronic myeloid leukaemia patients.

For a personalised approach in the prevention and management of TKI-associated toxicities, tailored assessment and early recognition of CV risk and CV events are cornerstones of the most convenient treatments. This study reinforces the need for close long-term follow-up that should be performed by a multidisciplinary team (general practitioner, haematologist, cardiologist). The target should not only be identification of the disease and specific drug-related toxicities but, also, cardiovascular and metabolic risk factors prior to the commencement of, as well as throughout, TKI therapy. This can result in the minimising of CV complications and increasing the quality of life and disease-specific survival.

## Figures and Tables

**Table 1:** Clinical features of 43 chronic myeloid leukaemia patients at the time of diagnosis.

	Imatinib (22 pts)	Nilotinib (12 pts)	Dasatinib (9 pts)	*p*-Value	Overall (43 pts)
Gender (male)	10 (45.4%)	11 (91.7%)	5 (55.5%)		26 (60.5%)
Age (years)				0.10	
Mean (SD)	57.7 (±14.8)	53.6 (±19.0)	56.3 (±10.0)		56.3 (±15.0)
Min–Max	22–81	23–76	38–70		22–81
BMI (kg/m^2^)				0.50	
Mean (SD)	28.1 (±4.9)	27.1 (±3.8)	29.0 (±5.5)		28.0 (±4.7)
Min–Max	17.7–38.7	21.5–32.9	23.1–37.0		17.7–38.7
Prior smoking	4 (18.2%)	3 (25.0%)	2 (22.2%)	0.89	9 (20.9%)
Prior DM	4 (18.2%)	1 (8.3%)	1 (11.1%)	0.70	6 (13.9%)
Prior hyperlipidaemia	2 (9.1%)	5 (41.7%)	4 (44.4%)	0.03	11 (25.6%)
Prior HTN	10 (45.4%)	5 (41.7%)	5 (55.5%)	0.81	20 (46.5%)
SBP before TKI (mmHg)				0.04	
Mean (SD)	128.4 (±16.1)	127.9 (±6.5)	126 (±12.2)		127.8 (±13.0)
Min–Max	100–170	120–140	105–140		100–170
DBP before TKI (mmHg)				0.49	
Mean (SD)	75.2 (±7.6)	74.33(±8.1)	70.0 (±7.5)		74.5 (±7.6)
Min–Max	60–90	60–90	60–80		60–90
Prior CAD	2 (9.1%)	0	0		2 (4.6%)
Prior arrhythmia	0	0	0		0
Prior LEAD	1 (4.5%)	0	0		1 (2.3%)
Prior HF	3 (13.6%)	2 (16.7%)	0	0.45	5 (11.6%)
Sokal				0.33	
Low	16 (72.7%)	5 (41.7%)	6 (66.7%)		27 (62.8%)
Intermediate	5 (22.7%)	5 (41.7%)	3 (33.3%)		13 (30.2%)
High	1 (4.5%)	2 (16.7%)	0		3 (7.0%)
Hasford					
Low	21 (95.4%)	11 (91.7%)	9 (100%)		41 (95.3%)
Intermediate	1 (4.5%)	1 (8.3%)	0		2 (4.6%)
High	0	0	0		0
Eutos long-term survival				0.02	
Low-risk group	10 (45.4%)	2 (16.7%)	4 (44.4%)		16 (37.2%)
Intermediate-risk group	11 (50.5%)	5 (41.7%)	5 (55.5%)		21 (48.8%)
High-risk group	1 (4.5%)	5 (41.7%)	0		6 (13.9%)

SD—standard deviation, BMI—body mass index, DM—diabetes mellitus, HTN—hypertension, SBP—systolic blood pressure, DBP—diastolic blood pressure, CAD—coronary artery disease, LEAD—lower extremity artery disease, HF—heart failure.

**Table 2:** Laboratory tests at the time of diagnosis.

Parameter	Imatinib (22 pts)	Nilotinib (12 pts)	Dasatinib (9 pts)	*p*-Value	Overall (43 pts)
WBC, ×10^9^/L				0.67	
Mean (SD)	109.6 (±84.3)	99.4 (±119.6)	177.8 (±207.1)		113.0 (±100.1)
Min–Max	17.5–292.0	15.8–397.0	14.9–640.0		14.9–640.0
Haemoglobin, g/dL				0.56	
Mean (SD)	11.9 (±2.2)	11.7 (±3.4)	12.8 (±2.2)		12.0 (±2.6)
Min–Max	6.1–14.8	4.6–16.7	10.3–15.8		4.6–16.7
Platelets, ×10^9^/L				0.004	
Mean (SD)	413.6 (±259.7)	306.4 (±344.7)	177.8 (±207.1)		375.2 (±283.6)
Min–Max	173.0–119.7	102.0–1360.0	14.9–640.0		14.9–1360.0
Cholesterol, mg/dL				0.03	
Mean (SD)	175.1 (±44.5)	196.9 (±39.6)	203.4 (±37.4)		187.1 (±42.8)
Min–Max	89.0–319.0	113.0–267.0	163.0–290.0		89.0–319.0
Triglyceride, mg/dL				0.51	
Mean (SD)	166.9 (±133.8)	112.3 (±40.3)	135.8 (±46.3)		145.2 (±101.8)
Min–Max	69.0–531.0	41.0–184.0	52.0–186.0		41.0–531.0
Blood glucose level, mg/dL				0.03	
Mean (SD)	97.1 (±10.8)	123.5 (±50.4)	99.7 (±15.3)		105.0 (±30.1)
Min–Max	79.0–117.0	90.0–277.0	84.0–130.0		79.0–277.0
BUN (mg/dl)				0.23	
Mean (SD)	40.6 (±16.1)	30.0 (±11.8)	35.1 (±8.7)		36.2 (±14.2)
Min–Max	25.7–94.0	13.0–45.0	23.0–49.0		13.0–94.0
Serum creatinine, mg/dL				0.37	
Mean (SD)	0.8 (±0.3)	0.6 (±0.4)	0.8 (±0.3)		0.7 (±0.3)
Min–Max	0.2–1.6	0.7–1.0	0.8–1.1		0.2–1.6
eGFR, mL/min/1.73 m^2^				0.45	
Mean (SD)	84.7 (±21.0)	79.7 (±27.5)	79.7 (±25.1)		82.3 (±23.3)
Min–Max	46.0–123.0	44.0–130.0	45.0–109.0		44.0–130.0

WBC—white blood cells, SD—standard deviation, BUN—blood urea nitrogen, eGFR–estimated glomerular filtration rate (CKD-EPI formula).

**Table 3:** Charlson Comorbidity Index of patients.

Charlson Comorbidity Index	Imatinib (*n*)	Nilotinib (*n*)	Dasatinib (*n*)	Overall (*n* (%))
2	5	5	2	12 (27.9%)
3	7	1	2	10 (23.2%)
4+	10	6	5	21 (48.8%)

**Table 4:** Systematic Coronary Risk Evaluation (SCORE) risk in chronic myeloid leukaemia (CML) patients.

Drug		SCORE Risk			
		Low	Moderate	High	Very High
**Imatinib**	Diagnosis time (22 pts)	5	6	1	10
	CML disease progression (22 pts)	4	7	1	10
**Nilotinib**	Diagnosis time (12 pts)	2	3	1	6
	CML disease progression (12 pts)	1	3	1	7
**Dasatinib**	Diagnosis time (9 pts)	0	5	3	1
	CML disease progression (9 pts)	0	5	2	2

**Table 5:** Re-evaluation of patient characteristics.

Parameter	Imatinib (22 pts)	Nilotinib (12 pts)	Dasatinib (9 pts)	*p*-Value	Overall (43 pts)
WBC, ×10^9^/L				<0.0001	
Mean (SD)	7.8 (±1.9)	9.1 (±2.3)	8.6 (±2.6)		8.3 (±2.2)
Min–Max	4.4–11.3	5.4–13.6	5.0–13.2		4.4–13.6
Haemoglobin, g/dL				0.05	
Mean (SD)	12.9 (±0.9)	13.2(±1.0)	13.3 (±1.1)		13.0 (±1.0)
Min–Max	11.5–14.7	11.4–14.8	11.9–15.4		11.4–15.4
Platelets, ×10^9^/L				0.42	
Mean (SD)	299.3 (±73.9)	249.0 (±96.0)	250.4 (±90.1)		275.3 (±85.5)
Min-Max	202.0–489.0	134.0–457.0	135.0–454.0		134.0–489.0
Cholesterol, mg/dL				0.04	
Mean (SD)	188.0 (±31.9)	229.0 (±48.8)	216.0 (±43.0)		205.0 (±42.9)
Min–Max	143.0–265.0	172.0–340.0	158.0–284.0		143.0–340.0
Triglyceride, mg/dL				0.05	
Mean (SD)	124.2 (±37.4)	146.2 (±33.1)	146.1 (±43.2)		135.0 (±38.3)
Min–Max	67.0–220.0	87.0–202.0	78.0–187.0		67.0–220.0
Blood glucose level, mg/dL				0.48	
Mean (SD)	99.3 (±8.8)	114.0 (±13.9)	98.9 (±12.9)		103.0 (±12.9)
Min–Max	82.0–118.0	97.0–145.0	78.0–123.0		78.0–145.0
Serum creatinine, mg/dL				0.55	
Mean (SD)	0.8 (±0.3)	1.0(±0.3)	0.9 (±0.2)		0.9 (±0.3)
Min–Max	0.5–1.6	0.7–1.8	0.6–1.2		0.5–1.8
eGFR, mL/min/1.73 m^2^				0.49	
Mean (SD)	89.4 (±25.2)	87.9 (±31.4)	86.2 (±23.6)		88.3 (±26.1)
Min–Max	34.0–127.0	37.0–135.0	49.0–129.0		34.0–135.0
SBP after TKI (mmHg)				0.0002	
Mean (SD)	142 (±25.4)	147.4 (±13.4)	138.1 (±16.9)		142.7 (±20.9)
Min–Max	95–200	130–174	114–170		95–200
DBP after TKI (mmHg)				0.002	
Mean (SD)	80.4 (±10.6)	83.2 (±9.6)	76.1 (±8.3)		80.3 (±10.0)
Min–Max	54–106	70–102	60–90		54–106

**Table 6:** Cardiovascular SCORE risk, cardiovascular, atherothrombotic events and effusions, in CML cohort.

Patient	Age (Years)	Gender	Drug	Dose at Event	CV Risk at Diagnostic Time	Event	Time to the Event (Months)
1	79	F	Imatinib First-line	400 mg o.d.	very high	STEMI	54
2	70	M	Nilotinib Second-line	400 mg b.i.d.	very high	Acute arterial occlusion	19
3	38	M	Nilotinib Second-line	400 mg b.i.d.	low	STEMI	54
4	68	M	Nilotinib Second-line	400 mg b.i.d.	very high	Arrhythmia	38
5	76	M	Nilotinib Second-line	400 mg b.i.d.	very high	Ischaemic stroke	28
6	70	M	Dasatinib Second-line	100 mg o.d.	very high	STEMI	38
						PH Moderate pleural effusion	50
						Moderate pericardial effusion	50
						Congestive HF	73
7	57	M	Dasatinib Second-line	100 mg o.d.	high	PH	48
						Moderate pericardial effusion. Moderate pleural effusion	48
8	56	M	Imatinib First-line	400 mg o.d.	high	Congestive HF	46
						HTN	60
9	44	M	Imatinib First-line	400 mg o.d.	low	Congestive HF	66
10	66	F	Imatinib First-line	400 mg o.d.	very high	Congestive HF	46
11	67	M	Imatinib First-line	400 mg o.d.	very high	HTN	36
12	62	F	Imatinib First-line	400 mg o.d.	very high	HTN	102
13	54	M	Imatinib First-line	400 mg o.d.	moderate	HTN	18
14	58	M	Nilotinib First-line	300 mg b.i.d.	very high	HTN	36
15	70	M	Nilotinib Second-line	400 mg b.i.d.	very high	HTN	29
16	38	M	Dasatinib Second-line	100 mg o.d.	moderate	HTN Small pericardial effusion	72
17	56	F	Dasatinib First-line	100 mg o.d.	moderate	Small pericardial effusion	18
18	48	F	Dasatinib Second-line	100 mg o.d.	moderate	Small pericardial effusion	90
19	24	M	Nilotinib First-line	300 mg b.i.d.	low	Small pericardial effusion	42
20	23	M	Nilotinib First-line	300 mg b.i.d.	low	Small pericardial effusion	29
21	57	M	Nilotinib Second-line	400 mg b.i.d.	high	Small pericardial effusion	30
22	67	M	Imatinib First-line	400 mg o.d.	very high	Small pericardial effusion	36
23	75	M	Imatinib First-line	400 mg o.d.	very high	Small pericardial effusion	114
24	53	F	Imatinib First-line	400 mg o.d.	moderate	Small pericardial effusion	26
25	68	F	Imatinib First-line	400 mg o.d.	very high	Small pericardial effusion	102

STEMI—acute myocardial infarction; PH—pulmonary hypertension; HF—heart failure; HTN—hypertension.

## References

[1] Hoglund M., Sandin F., Simonsson B. (2015). Epidemiology of chronic myeloid leukaemia: An update. Ann. Hematol..

[2] Medeiros B.C., Possick J., Fradley M. (2018). Cardiovascular, pulmonary, and metabolic toxicities complicating tyrosine kinase inhibitor therapy in chronic myeloid leukemia: Strategies for monitoring, detecting, and managing. Blood Rev..

[3] Caldemeyer L., Dugan M., Edwards J., Akard L.P. (2016). Long-term side effects of tyrosine kinase inhibitors in chronic myeloid leukemia. Curr. Hematol. Malignacy Rep..

[4] Chaar M., Kamta J., Ait-Oudhia S. (2018). Mechanisms, monitoring, and management of tyrosine kinase inhibitors–associated cardiovascular toxicities. OncoTargets Ther..

[5] Valent P., Hadzijusufovic E., Hörmann G., Füredera W., Schernthaner G.-H., Sperr W.R., Kirchmair R., Wolf D. (2017). Risk factors and mechanisms contributing to TKI-induced vascular events in patients with CML. Leuk. Res..

[6] Steegmann J.L., Baccarani M., Breccia M., Casado L.F., García-Gutiérrez V., Hochhaus A., Kim D.-W., Kim T.D., Khoury H.J., Le Coutre P. (2016). European LeukemiaNet recommendations for the management and avoidance of adverse events of treatment in chronic myeloid leukaemia. Leukemia.

[7] Cortes J., Khoury H.J., Kantarjian H., Brümmendorf T.H., Mauro M.J., Matczak E., Pavlov D., Aguiar J.M., Fly K.D., Dimitrov S. (2016). Long-term evaluation of cardiac and vascular toxicity in patients with Philadelphia chromosome-positive leukemias treated with bosutinib. Am. J. Hematol..

[8] Agarwal M., Thareja N., Benjamin M., Akhondi A., Mitchell G.D. (2018). Tyrosine kinase inhibitor-induced hypertension. Curr. Oncol. Rep..

[9] Weatherald J., Chaumais M.-C., Montani D. (2017). Pulmonary arterial hypertension induced by tyrosine kinase inhibitors. Curr. Opin. Pulm. Med..

[10] Özgür Yurttaş N., Eskazan A.E. (2018). Dasatinib-induced pulmonary arterial hypertension. Br. J. Clin. Pharmacol..

[11] Breccia M., Molica M., Zacheo I., Serrao A., Alimena G. (2014). Application of systematic coronary risk evaluation chart to identify chronic myeloid leukemia patients at risk of cardiovascular diseases during nilotinib treatment. Ann. Hematol..

[12] Piepoli M.F., Hoes A.W., Agewall S., Albus C., Brotons C., Catapano A.L., Cooney M.T., Corrà U., Cosyns B., Deaton C. (2016). 2016 European guidelines on cardiovascular disease prevention in clinical practice: The Sixth Joint Task Force of the European Society of Cardiology and Other Societies on Cardiovascular Disease Prevention in Clinical Practice (constituted by representatives of 10 societies and by invited experts) developed with the special contribution of the European Association for Cardiovascular Prevention & Rehabilitation (EACPR). Eur. Heart J..

[13] Caocci G., Mulas O., Abruzzese E., Iurlo A., Annunziata M., Orlandi E.M., Galimberti S., Binotto G., Sgherza N., Luciano L. (2019). Incidence and evaluation of predisposition to cardiovascular toxicity in chronic myeloid leukemia patients treated with bosutinib in the real-life practice. Ann. Hematol..

[14] Caocci G., Mulas O., Abruzzese E., Luciano L., Iurlo A., Attolico I., Castagnetti F., Galimberti S., Sgherza N., Bonifacio M. (2019). Arterial occlusive events in chronic myeloid leukemia patients treated with ponatinib in the real-life practice are predicted by the Systematic Coronary Risk Evaluation (SCORE) chart. Hematol Oncol..

[15] Caocci G., Mulas O., Annunziata M., Luciano L., Bonifacio M., Orlandi E.M., Pregno P., Galimberti S., Russo Rossi A., Abruzzese E. (2018). Cardiovascular toxicity in patients with chronic myeloid leukemia treated with second-generation tyrosine kinase inhibitors in the real-life practice: Identification of risk factors and the role of prophylaxis. Am. J. Hematol..

[16] Baccarani M., Deininger M.W., Rosti G., Hochhaus A., Soverini S., Apperley J.F., Cervantes F., Clark R.E., Cortes J.E., Guilhot F. (2013). European LeukemiaNet recommendations for the management of chronic myeloid leukemia: 2013. Blood.

[17] Jain P., Kantarjian H., Boddu P.C., Nogueras-González G.M., Verstovsek S., Garcia-Manero G., Borthakur G., Sasaki K., Kadia T.M., Sam P. (2019). Analysis of cardiovascular and arteriothrombotic adverse events in chronic-phase CML patients after frontline TKIs. Blood Adv..

[18] Cirmi S., El Abd A., Letinier L., Navarra M., Salvo F. (2020). Cardiovascular Toxicity of Tyrosine Kinase Inhibitors Used in Chronic Myeloid Leukemia: An Analysis of the FDA Adverse Event Reporting System Database (FAERS). Cancers (Basel).

[19] Stevens P.E. (2013). Evaluation and management of chronic kidney disease: Synopsis of the kidney disease: Improving global outcomes 2012 clinical practice guideline. Ann. Intern. Med..

[20] Williams B., Mancia G., Spiering W., Rosei E.A., Azizi M., Burnier M., Clement D.L., Coca A., De Simone G., Dominiczak A.F. (2018). 2018 ESC/ESH guidelines for the management of arterial hypertension. Eur. Hear. J..

[21] Ponikowski P., Voors A.A., Anker S.D., Bueno H., Cleland J.G.F., Coats A.J.S., Falk V., González-Juanatey J.R., Harjola V.P., Jankowska E.A. (2016). 2016 ESC Guidelines for the diagnosis and treatment of acute and chronic heart failure: The Task Force for the diagnosis and treatment of acute and chronic heart failure of the European Society of Cardiology (ESC). developed with the special contribution of the Heart Failure Association (HFA) of the ESC. Eur. J. Heart Fail..

[22] Plana J.C., Galderisi M., Barac A., Ewer M.S., Ky B., Scherrer-Crosbie M., Ganame J., Sebag I.A., Agler D.A., Badano L.P. (2014). Expert consensus for multimodality imaging evaluation of adult patients during and after cancer therapy: A report from the American Society of Echocardiography and the European Association of Cardiovascular Imaging. Eur. Heart J. Cardiovasc. Imaging.

[23] Aboyans V., Ricco J.-B., Bartelink M.-L., Björck M., Brodmann M., Cohnert T., Collet J.-P., Czerny M., De Carlo M., Debus S. (2017). 2017 ESC Guidelines on the Diagnosis and Treatment of Peripheral Arterial Diseases, in collaboration with the European Society for Vascular Surgery (ESVS). Eur. Hear. J..

[24] Mach F., Baigent C., Catapano A.L., Koskinas K.C., Casula M., Badimon L., Chapman M.J., De Backer G.G., Delgado V., Ference B.A. (2020). ESC Scientific Document Group. 2019 ESC/EAS Guidelines for the management of dyslipidaemias: Lipid modification to reduce cardiovascular risk. Eur. Heart J..

[25] Hochhaus A., Baccarani M., Silver R.T., Schiffer C., Apperley J.F., Cervantes F., Clark R.E., Cortes J.E., Deininger M.W., Guilhot F. (2020). European LeukemiaNet 2020 recommendations for treating chronic myeloid leukemia. Leukemia.

[26] Uemura M., Imataki O., Kawachi Y., Kawakami K., Hoshijima Y., Matsuoka A., Kadowaki N. (2016). Charlson comorbidity index predicts poor outcome in CML patients treated with tyrosine kinase inhibitor. Int. J. Hematol..

[27] Breccia M., Latagliata R., Stagno F., Luciano L., Gozzini A., Castagnetti F., Fava C., Cavazzini F., Annunziata M., Rossi A.R. (2011). Charlson comorbidity index and adult comorbidity evaluation-27 scores might predict treatment compliance and development of pleural effusions in elderly patients with chronic myeloid leukemia treated with second-line dasatinib. Haematologica.

[28] Jabbour E., Kantarjian H.M. (2018). Chronic myeloid leukemia: 2018 update on diagnosis, therapy and monitoring. Am. J. Hematol..

[29] Ross D.M., Arthur C., Burbury K., Ko B.S., Mills A.K., Shortt J., Kostner K. (2018). Chronic myeloid leukaemia and tyrosine kinase inhibitor therapy: Assessment and management of cardiovascular risk factors. Intern. Med. J..

[30] Osada Y., Arakaki H., Takanashi S., Ito C., Aisa Y., Nakazato T. (2017). Association between a cardiovascular disease risk assessment and the molecular response to tyrosine kinase inhibitors in chronic-phase chronic myeloid leukemia patients. Med. Oncol..

[31] Ashry N.A., Abdеlaziz R.R., Suddеk G.M. (2020). The potential effect of imatinib against hypercholesterolemia induced atherosclerosis, endothelial dysfunction and hepatic injury in rabbits. Life Sci..

[32] Haguet H., Douxfils J., Chatelain C., Graux C., Mullier F., Dogné J.-M. (2018). BCR-ABL tyrosine kinase inhibitors: Which mechanism(s) may explain the risk of thrombosis?. TH Open.

[33] Hadzijusufovic E., Albrecht-Schgoer K., Huber K., Hoermann G., Grebien F., Eisenwort G., Schgoer W., Herndlhofer S., Kaun C., Theurl M. (2017). Nilotinib-induced vasculopathy: Identification of vascular endothelial cells as a primary target site. Leukemia.

[34] Alhawiti N., Burbury K.L., Kwa F.A., O’Malley C.J., Shuttleworth P., Alzard M., Hamadi A., Grigg A.P., Jackson D.E. (2016). The tyrosine kinase inhibitor, nilotinib potentiates a prothrombotic state. Thromb. Res..

[35] Dahlén T., Edgren G., Lambe M., Höglund M., Björkholm M., Sandin F., Själander A., Richter J., Olsson-Strömberg U., Ohm L. (2016). Swedish CML Group and the Swedish CML Register Group. Ann. Intern. Med..

[36] Góra-Tybor J., Medras E., Calbecka M., Kołkowska-Leśniak A., Ponikowska-Szyba E., Robak T., Jamroziak K. (2015). Real-life comparison of severe vascular events and other non-hematological complications in patients with chronic myeloid leukemia undergoing second-line nilotinib or dasatinib treatment. Leuk. Lymphoma.

[37] Quintás-Cardama A., Kantarjian H., Cortes J. (2012). Nilotinib—Associated vascular events. Clin. Lymphoma Myeloma Leuk..

[38] Novo G., Di Lisi D., Bronte E., Macaione F., Accurso V., Badalamenti G., Rinaldi G., Siragusa S., Novo S., Russo A. (2020). Cardiovascular toxicity in cancer patients treated with tyrosine kinase inhibitors: A real-world single-center experience. Oncology.

[39] Levato L., Cantaffa R., Kropp M.G., Magro D., Piro E., Molica S. (2013). Progressive peripheral arterial occlusive disease and other vascular events during nilotinib therapy in chronic myeloid leukemia: A single institution study. Eur. J. Haematol..

[40] Aghel N., Lipton J.H., Atenafu E.G., Kim D., Dong H., Delgado D.H. (2017). Cardiovascular events after exposure to nilotinib in chronic myeloid leukemia: Long-term follow-up. Clin. Lymphoma Myeloma Leuk..

[41] Franklin M., Burns L., Perez S., Yerragolam D., Makenbaeva D. (2017). Incidence of type 2 diabetes mellitus and hyperlipidemia in patients prescribed dasatinib or nilotinib as first or second-line therapy for chronic myelogenous leukemia in the US. Curr. Med. Res. Opin..

[42] Molica M., Scalzulli E., Colafigli G., Fegatelli D.A., Massaro F., Latagliata R., Foà R., Breccia M. (2018). Changes in estimated glomerular filtration rate in chronic myeloid leukemia patients treated front line with available TKIs and correlation with cardiovascular events. Ann. Hematol..

[43] Assunção P.M., Lana T.P., Delamain M.T., Duarte G.O., Zulli R., Lorand-Metze I., De Souza C.A., De Paula E.V., Pagnano K.B. (2019). Cardiovascular risk and cardiovascular events in patients with chronic myeloid leukemia treated with tyrosine kinase inhibitors. Clin. Lymphoma Myeloma Leuk..

[44] Kozako T., Soeda S., Yoshimitsu M., Arima N., Kuroki A., Hirata S., Tanaka H., Imakyure O., Tone N., Honda S.-I. (2016). Angiotensin II type 1 receptor blocker telmisartan induces apoptosis and autophagy in adult T-cell leukemia cells. FEBS Open Bio.

[45] Schoepf A.M., Salcher S., Obexer P., Gust R. (2019). Overcoming imatinib resistance in chronic myelogenous leukemia cells using non-cytotoxic cell death modulators. Eur. J. Med. Chem..

[46] Schoepf A.M., Salcher S., Hohn V., Veider F., Obexer P., Gust R. (2020). Synthesis and characterization of telmisartan-derived cell death modulators to circumvent imatinib resistance in chronic myeloid leukemia. ChemMedChem.

[47] Varga A., Farczadi L., Vlase L., Primejdie D.P., Carasca E., Tilea I. (2015). Liquid chromatography tandem mass spectrometry simultaneous determination of amlodipine and telmisartan in human plasma for therapeutic drug monitoring. Rev. Chim..

[48] Rea D., Ame S., Charbonnier A., Coiteux V., Cony-Makhoul P., Escoffre-Barbe M., Etienne G., Gardembas M., Guerci-Bresler A., Legros L. (2016). Recommandations 2015 du France Intergroupe des Leucémies Myéloïdes Chroniques pour la gestion du risque d’événements cardiovasculaires sous nilotinib au cours de la leucémie myéloïde chronique. Bull. Cancer..

[49] Granatowicz A., Piatek C.I., Moschiano E., El-Hemaidi I., Armitage J.D., Akhtari M. (2015). An overview and update of chronic myeloid leukemia for primary care physicians. Korean J. Fam. Med..

